# Impact of Early Rehabilitation Initiation on Walking Recovery in Critically Ill Patients

**DOI:** 10.7759/cureus.88280

**Published:** 2025-07-19

**Authors:** Ryuji Yoshinaga, Narumi Yamada, Ryo Kozu

**Affiliations:** 1 Department of Physical Therapy Science, Nagasaki University Graduate School of Biomedical Sciences, Nagasaki, JPN; 2 Department of Rehabilitation Medicine, National Hospital Organization Ureshino Medical Center, Ureshino, JPN; 3 Department of Clinical Research Center, National Hospital Organization Nagasaki Medical Center, Omura, JPN; 4 Department of Emergency Medicine, National Hospital Organization Ureshino Medical Center, Nagasaki, JPN

**Keywords:** critically ill patients, intensive care unit, mechanical ventilation, rehabilitation, walking recovery

## Abstract

Purpose

Predicting walking recovery in critically ill patients, including the timing of early rehabilitation, remains unclear. This study aimed to identify clinical factors, particularly the timing of first sitting on the edge of the bed, that are associated with walking recovery in critically ill patients.

Methods

We retrospectively analyzed data from mechanically ventilated patients (≥48 hours) admitted to the intensive care unit (ICU) of a tertiary-care hospital. Only patients who survived until hospital discharge were included. Walking recovery was defined as achieving a Functional Ambulation Categories (FAC) score ≥ 3, indicating independent walking on level ground with supervision for safety.

Results

A total of 121 patients were included. The median age was 73 years, 83 (68.6%) were male, the mean Acute Physiology and Chronic Health Evaluation II (APACHE II) score was 18.9 ± 6.5, and the median duration of mechanical ventilation was seven days. Cox proportional-hazards regression identified three independent predictors of walking recovery: younger age (hazard ratio (HR) 0.98; 95% CI 0.97-1.00; p = 0.016), shorter duration of mechanical ventilation (HR 0.97; 95% CI: 0.95-1.00; p = 0.049), and sitting on the edge of the bed within 13 days (HR 0.11; 95% CI 0.52-0.91; p < 0.001). Receiver operating characteristic (ROC) analysis identified day 13 as the optimal cutoff for sitting, with an area under the curve (AUC) of 0.76, sensitivity of 81.6%, and specificity of 67.4% for predicting walking recovery. Gray’s test further demonstrated that initiating sitting after 13 days significantly reduced the probability of walking recovery compared to earlier initiation (p < 0.001).

Conclusion

Achieving a sitting position at the edge of the bed by day 13 may serve as a clinically relevant marker for timely rehabilitation initiation and improved walking recovery in critically ill patients. However, the timing for initiating sitting on the edge of the bed should be validated through future prospective multicenter studies.

## Introduction

Critically ill patients requiring mechanical ventilation in intensive care units (ICU) often experience immobility-related physical decline [1]. Early rehabilitation is recommended for critically ill patients requiring mechanical ventilation for 24 hours or more to prevent such deterioration [2]. Studies have shown that rehabilitation can improve walking ability at discharge in critically ill patients [3,4]. The proportion of patients achieving independent walking at discharge or transfer ranges from 38% to 64% [5,6], indicating that nearly half may experience mobility limitations. These findings emphasize the importance of identifying clinical indicators to guide early rehabilitation for walking recovery.

Recovery of walking ability is a key factor associated with discharge from the hospital to home [4,7,8]. Walking ability reflects not only physical function but also the capacity to perform basic activities of daily living independently, such as toileting and transferring. Therefore, patients who regain walking ability by discharge are more likely to return home [5]. Previous studies have demonstrated that the recovery of physical function in critically ill patients requiring prolonged mechanical ventilation is influenced by factors such as age, comorbidities, duration of sedation and mechanical ventilation, sepsis, and acute respiratory distress syndrome [9-12]. Furthermore, among factors related to early rehabilitation, such as rehabilitation programs and progression, the time spent walking during hospitalization has been reported to increase the likelihood of regaining walking ability [13]. These findings suggest that, with appropriate intervention, critically ill patients may have the potential to recover walking ability. However, in clinical practice, some patients may be unable to walk or even stand, often resulting in prolonged bed rest and the necessity for transfer to other hospitals. Thus, during early rehabilitation, the timing of achieving a sitting position before walking may serve as a valuable clinical indicator.

Functional recovery in critical care follows a hierarchical process, where early postural control (e.g., sitting balance) precedes higher-level mobility like walking [14]. Sitting on the edge of the bed is an early milestone in ICU mobilization and a benchmark in progressive mobility protocols [15]. It challenges postural control and neuromuscular coordination, indicating functional readiness. However, its timing in relation to walking recovery remains unclear in the literature.

Therefore, we hypothesized that the timing of initiating sitting on the edge of the bed would be associated with walking recovery in critically ill patients undergoing mechanical ventilation. This study aimed to examine whether the timing of initiating sitting on the edge of the bed influences walking recovery in critically ill patients.

## Materials and methods

Study design and patients

This retrospective cohort study was conducted at a single tertiary-care hospital. The study included patients who underwent mechanical ventilation during ICU admission at the National Hospital Organization Nagasaki Medical Center in Nagasaki, Japan, between April 2016 and March 2018. The eligibility criteria were age 18 years or older, continuous mechanical ventilation for at least 48 hours, ICU stay for at least 72 hours, and physician-prescribed rehabilitation. These thresholds were set to ensure the inclusion of patients who had sufficient exposure to intensive care and rehabilitation services. Exclusion criteria included difficulty walking before hospitalization, neurological disorders such as stroke, epilepsy, and neuromuscular diseases managed by neurology or neurosurgery, in-hospital death, amputation of the upper and lower limbs, spinal cord injury, and pelvic and/or lower limb fractures. The screening of patient eligibility was performed by the first author through a retrospective review of electronic medical records. As this was a retrospective study, blinding the screeners to the study outcomes was not applicable. Patients with pre-hospital walking difficulties were defined as those who were wheelchair-dependent before admission, as documented in the medical records. This study was approved by the Ethics Committee of the Institutional Review Board of the National Hospital Organization Nagasaki Medical Center (approval no. 30039). An opt-out method was employed for informed consent, and the study details were made publicly accessible via the hospital’s website, in compliance with the Ethics Committee's requirements. The sample size was determined based on the number of eligible patients admitted to the ICU during the study period, and no formal sample size calculation was performed.

Study setting and rehabilitation program

This study was performed in the ICU of the National Hospital Organization Nagasaki Medical Center, a tertiary hospital. The total number of beds in the ICU was 16, including the burn unit. The rehabilitation program was administered by trained physical therapists (PT) or occupational therapists (OT) in one to two sessions per day, five days a week. The rehabilitation program included respiratory physiotherapy, passive and active range-of-motion exercises, early mobilization, sit-to-stand training, standing, and marching in place, along with progressive mobilization. The program was initiated in daily consultation with the ICU physician and PT. In the ICU, sitting on the edge of the bed and standing exercises were initiated after the physician ruled out venous thromboembolism on ultrasound examination of the lower extremities. After ICU discharge, ultrasound evaluation was not consistently performed in all patients. During the study period, standardized criteria for starting or discontinuing rehabilitation were not established at our institution. The decision to initiate sitting at the edge of the bed was typically made based on the judgment of the primary physician. The rehabilitation program was not based on a standardized protocol but was individualized according to each patient’s condition and the attending physician’s clinical judgment. All patients continued receiving standard rehabilitation sessions from the same PT or OT after discharge from the ICU.

Data collection

We retrospectively collected clinical data from the medical records of study participants. At admission, we recorded the following variables: age, gender, body mass index (BMI), diagnosis, Charlson Comorbidity Index (CCI) [16], Sequential Organ Failure Assessment (SOFA) score [17], Acute Physiology and Chronic Health Evaluation II (APACHE II) score [18], and walking ability before admission. Data collected up to ICU discharge included the presence or absence of surgery, duration of invasive mechanical ventilation, deep sedation period (defined as Richmond Agitation-Sedation Scale score < -2) [19], the number of days from ICU admission to ultrasound examination of the lower extremities to rule out lower extremity deep vein thrombosis (DVT), minimum partial pressure of oxygen (PaO_2_)/fraction of inspired oxygen (FIO_2_) (P/F) ratio, presence of tracheostomy, and occurrence of ventilator-associated pneumonia (VAP). VAP was defined according to the Centers for Disease Control and Prevention criteria as pneumonia developing after more than 48 hours of mechanical ventilation [20], and blood glucose levels exceeded 200 mg/dL at least twice in one day, in accordance with previously established criteria [21]. This was treated as a binary variable (yes/no), indicating whether the criterion was met at least once during the ICU stay. At ICU discharge, we investigated the proportion of patients who had commenced sitting, standing, walking with a walker, and walking with a cane or unassisted, as well as the number of days until each activity was initiated. We also examined the proportion of patients discharged home and the length of hospital stay.

Outcomes

The main outcome of this study was the recovery of walking ability, defined as a Functional Ambulation Categories (FAC) ≥ 3 [13,22]. The FAC comprises six walking levels based on the amount of walking assistance (ranging from 0 to five points) [23] and allows walking ability to be evaluated objectively. A score of 0 represents difficulty walking, and a score of two indicates that one person is required to maintain balance to prevent falls when walking on level ground and to provide continuous or intermittent physical assistance to assist movement. An FAC ≥ 3 indicates the ability to walk on level ground without physical assistance; however, one person is required for proximal monitoring to ensure safety because of factors such as poor judgment, cardiac function problems, and the need for verbal instructions to perform movements. The occurrence of the primary event was defined as the earliest day on which the patient was able to regain walking ability without physical assistance from a physiotherapist while using a walking aid (such as a walker or cane). In our cohort, all patients used wheeled walkers as mobility aids during walking. The FAC was selected as the primary outcome measure for its simplicity, reliability, and feasibility in evaluating walking ability in critically ill patients, especially in the early recovery phase when many patients are unable to perform more complex tests such as the Six-Minute Walk Test or Timed Up and Go. Additionally, because this was a retrospective study, the FAC was chosen as it was consistently documented in the clinical records, allowing for standardized and objective assessment across patients. FAC scores were retrospectively extracted from standardized rehabilitation records, which were documented daily by licensed PTs responsible for each patient's rehabilitation. These scores were typically recorded during functional gait training sessions in the ward, based on direct observation of the patient's walking performance. This ensured consistency and objectivity in outcome classification across patients.

Statistical analysis

Continuous variables are summarized as mean ± standard deviation or median and interquartile range (IQR). The data were tested for normal distribution using the Shapiro-Wilk test. Categorical variables are described as frequencies and percentages. The Mann-Whitney U test was used for non-normally distributed variables. Comparisons between the recovery of walking ability group (walking group) and the non-recovery of walking ability group (non-walking group) were conducted using an unpaired t-test or Mann-Whitney test for continuous variables and the chi-square (χ2) test for categorical variables.

We used Cox proportional hazard regression analysis to examine the relationship between recovery of walking ability and relevant factors. The dependent variable was time-to-event from the start of mechanical ventilation to the recovery of walking ability, with an FAC ≥ 3. Independent variables included those with p < 0.2 from the results of the univariate analysis and clinically important variables such as age, severity of illness, and comorbidities, which have been reported to be associated with walking recovery in previous studies [5,6,12]. As this was an exploratory study, independent variables with p < 0.2 in the univariate analysis were included in the model to reduce the risk of missing potential confounders with clinical relevance. After confirming the multicollinearity among the independent variables, variable selection was performed using the forced entry method. Multicollinearity among independent variables was assessed using the variance inflation factor (VIF), with a VIF ≥ 10 considered indicative of multicollinearity. Censoring was defined as the number of days until transfer or discharge. When an extracted independent variable was a continuous variable, the optimal cutoff value for predicting walking recovery was determined using a receiver operating characteristic (ROC) curve, based on the classification into walking and non-walking groups, and the variable was then treated as a binary variable. This transformation was performed to facilitate clinical interpretation and to define a practical threshold, particularly for early rehabilitation milestones such as the timing of first sitting on the edge of the bed.

We classified the extracted independent factors into two groups based on walking recovery status: the walking group (patients with FAC ≥ 3) and the non-walking group (patients without walking recovery), and used the Kaplan-Meier method for comparison until the recovery of walking ability. The Gray’s test was used for comparison between groups. Although competing risks were not considered, this test was employed to facilitate graphical visualization of walking recovery. All statistical analyses were conducted using EZR (Easy R), version 1.54 (Saitama Medical Center, Jichi Medical University, Saitama, Japan), which is a graphical user interface for R (The R Foundation for Statistical Computing, Vienna, Austria, version 4.2.1) and functions as a modified version of R Commander designed for medical statistics [24]. A p-value < 0.05 was considered statistically significant.

## Results

Patient characteristics

**Figure 1:**
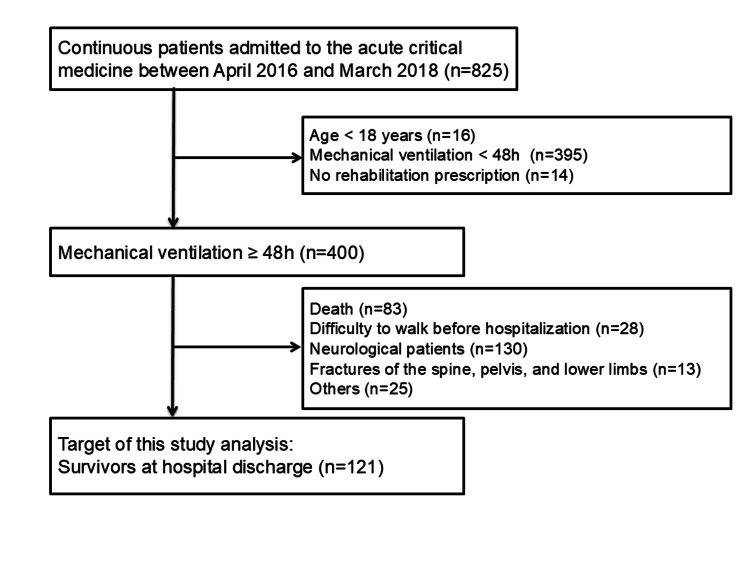
Flow diagram of the patient selection process

During the study period, 825 consecutive patients were admitted to the ICU. Of these, 400 met the eligibility criteria. After applying the exclusion criteria, 279 patients were excluded. Consequently, a total of 121 patients who underwent mechanical ventilation for ≥ 48 h and survived until discharge were included in the final analysis (Figure 1). The median age was 73 years, the mean APACHE II score was 18.9 ± 6.5, the median duration of mechanical ventilation was seven days, and the median deep sedation period was six days. The most common diagnoses were cardiovascular diseases in 36 (29.8%) and respiratory diseases in 31 (25.6%) patients. Of the 121 patients, 91 (75.1%) regained walking ability by discharge or transfer, while 30 (24.9%) had not. During the ICU stay, DVT was observed in nine (7.4%) patients. The walking independence rate was 60.6 (100 cases per person-month).

Univariate analysis (Table 1) revealed significant differences between the walking and non-walking groups in CCI, SOFA score, APACHE II score, duration of mechanical ventilation, deep sedation period, tracheostomy, hyperglycemia, time to sitting initiation, ICU stay, and hospital stay. Furthermore, significant differences were observed between the walking and non-walking groups in several rehabilitation-related variables. Compared to the walking recovery group, the non-walking group experienced significant delays in the initiation of sitting on the edge of the bed (10 vs. 22 days, p<0.001), standing (11 vs. 23 days, p<0.001), and walker-assisted walking (13 vs. 57 days, p<0.001). Sitting on the edge of the bed was implemented in all 121 patients, whereas standing, walker-assisted walking, and walking with walking aids were not achieved in some patients. 

**Table 1:** Characteristics of the 121 patients who underwent mechanical ventilation in the ICU Mean ± standard deviation, median (25th–75th percentile), or number; p-values were calculated using the Student’s t-test for normally distributed continuous variables, the Mann–Whitney U test for non-normally distributed continuous variables, and the chi-square test or Fisher’s exact test for categorical variables, as appropriate. The corresponding test statistics (t, U, or χ² values) are reported in the “Test statistic” column. Walking: a recovery of walking ability group; Non-walking: a non-recovery of walking ability group; ICU: intensive care unit; SOFA: Sequential Organ Failure Assessment; APACHE: Acute Physiology and Chronic Health Evaluation; RRT: renal-replacement therapy; IABP: intra-aortic balloon pumping; PCPS: percutaneous cardiopulmonary support; VAP: ventilator-associated pneumonia; VTE: venous thromboembolism; GNRI: Geriatric Nutritional Risk Index

Parameters		Total (n = 121)	Walking (n = 91)	Non-Walking (n = 30)	Test statistic	p-value
ICU admission						
Age (yrs)		73.0 (64.0-80.0)	72.0 (63.0-79.0)	78.0 (68.0-85.3)	U = 1662	0.075
Male Sex, n (%)		83 (68.6%)	62 (68.1%)	21 (70.0%)	χ² = 0	1
Body mass index (kg/m^2^)		21.1 (19.2-24.1)	21.5 (19.2-24.7)	20.6 (19.9-23.1)	U = 1185	0.279
ICU admission diagnosis, n (%)					χ² = 4.7	0.386
Cardiovascular		36 (29.8%)	30 (33.0%)	6 (20.0%)		
Respiratory (including pneumonia)		31 (25.6%)	20 (22.0%)	11 (36.7%)		
Gastrointestinal		30 (24.8%)	22 (24.2%)	8 (26.7%)		
Trauma		15 (12.4%)	13 (14.3%)	2 (6.7%)		
Sepsis, nonpulmonary		6 (5.0%)	4 (4.4%)	2 (6.7%)		
Other		3 (2.5%)	2 (2.2%)	1 (3.3%)		
Charlson comorbidity index		1.0 (0.0-2.0)	1.0 (0.0-2.0)	1.0 (0.0-2.8)	U = 1731	0.02
APACHE II score		18.9 ± 6.5	18.1 ± 6.4	21.2 ± 6.2	t = -2.3	0.024
SOFA score at ICU admission		6.0 (5.0-8.0)	6.0 (4.0-8.0)	7.0 (6.0-10.0)	U = 1805	0.008
During ICU stay						
Duration of mechanical ventilation (day)		7.0 (4.0-12.0)	6.0 (4.0-10.0)	12.0 (7.5-22.0)	U = 1988	<0.001
PaO_2_/F_I_O_2_		247 (167-333)	250 (188-368)	174 (125-277)	U = 874	0.005
Operations, n (%)		64 (52.9%)	49 (53.8%)	15 (50.0%)	χ² = 0.02	0.833
Deep sedation period (day)		6.0 (4.0-10.0)	8.5 (5.0-14.0)	5.0 (3.5-9.0)	U = 1733	0.027
Tracheotomy, n (%)		33 (27.3%)	16 (17.6%)	17 (56.7%)	χ² =15.5	<0.001
RRT, n (%)		14 (11.6%)	10 (11.0%)	4 (13.3%)	χ² = 0	0.746
IABP and/or PCPS, n (%)		5 (4.1%)	5 (5.5%)	0 (0.0%)	χ² = 0.6	0.331
VAP, n (%)		9 (7.4%)	6 (6.6%)	3 (10.0%)	χ² = 0.05	0.688
VTE, n (%)		9 (7.4%)	6 (6.6%)	3 (10.0%)	χ² = 0.05	0.688
Corticosteroids, n (%)		17 (14.0%)	11 (12.1%)	6 (20.0%)	χ² = 0.61	0.362
Hyperglycemia >200mg/dl, n (%)		49 (40.5%)	33 (36.3%)	16 (53.3%)	χ² = 2.07	0.133
GNRI		87.5 ± 13.1	88.3 ± 13.4	85.1 ± 12.2	t = -1.1	0.256
Days until ultrasound of lower limb		11.0 (7.0-16.0)	8.0 (6.0-11.0)	17.0 (13.3-23.8)	U = 582	<0.001
ICU length of stay (days)		13.0 (9.0-20.0)	11.0 (8.0-17.0)	19.0 (13.3-32.8)	U = 1958	<0.001
Rehabilitation						
Rehabilitation start days in ICU		4.0 (2.0-6.0)	4.0 (3.0-6.0)	4.5 (2.0-8.0)	U = 1441	0.646
Sitting on the edge, n (%)		121 (100%)	91 (100%)	30 (100%)	—	—
Sitting on the edge (day)		11.0 (8.0-22.0)	10.0 (7.0-14.0)	22.0 (14.0-30.0)	U = 2104	<0.001
Standing, n (%)		115 (95.0%)	91 (100.0%)	24 (80.0%)	χ² = 15.1	<0.001
Standing (day)		12.0 (9.0-22.0)	11.0 (8.0-16.0)	23.0 (15.0-32.5)	U = 1700	<0.001
Walker walking, n (%)		100 (82.6%)	91 (100.0%)	9 (30.0%)	χ² = 72.3	<0.001
Walker walking (day)		14.0 (10.0-30.8)	13.0 (10.0-19.5)	57.0 (49.0-69.0)	U = 684	0.001
Walking with walking aids or a hospital drip, n (%)		76 (62.8%)	76 (83.5%)	0 (0.0%)	χ² = 63.8	<0.001
Rehabilitation session (session/days)		1.3 (1.1-1.6)	1.3 (1.2-1.6)	1.5 (1.1-1.6)	U = 1455	0.587
Discharge to home, n (%)		32 (26.4%)	32 (35.2%)	0 (0.0%)	χ² = 15.0	<0.001
Hospital length of stay (days)		38.0 (27.0-64.0)	33.0 (25.0-56.5)	54.5 (37.3-93.5)	U = 1932	0.001

Rehabilitation indicators for recovery of walking ability

**Figure 2:**
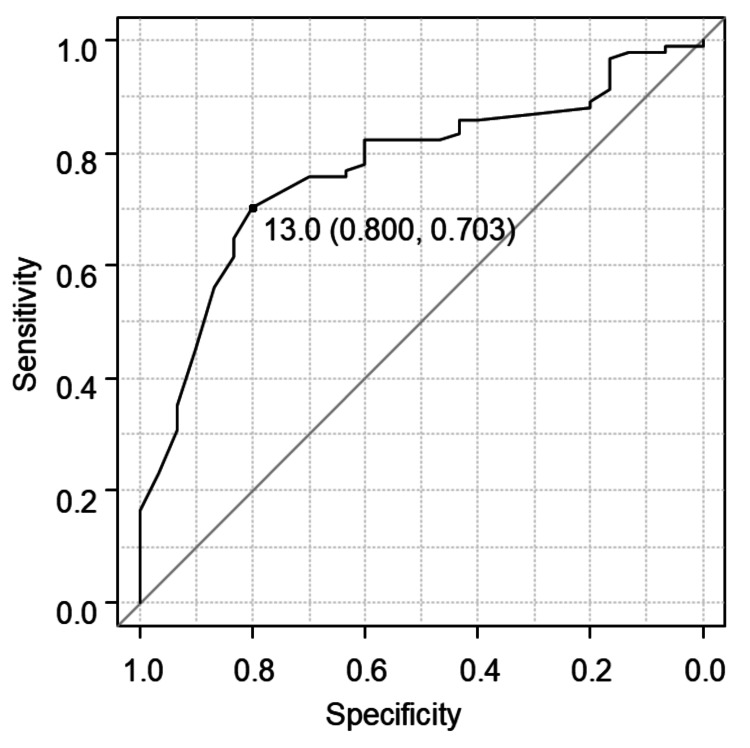
Receiver operating characteristic (ROC) curve analysis to identify the cutoff point for initiating edge-of-bed sitting in relation to walking ability recovery

**Figure 3:**
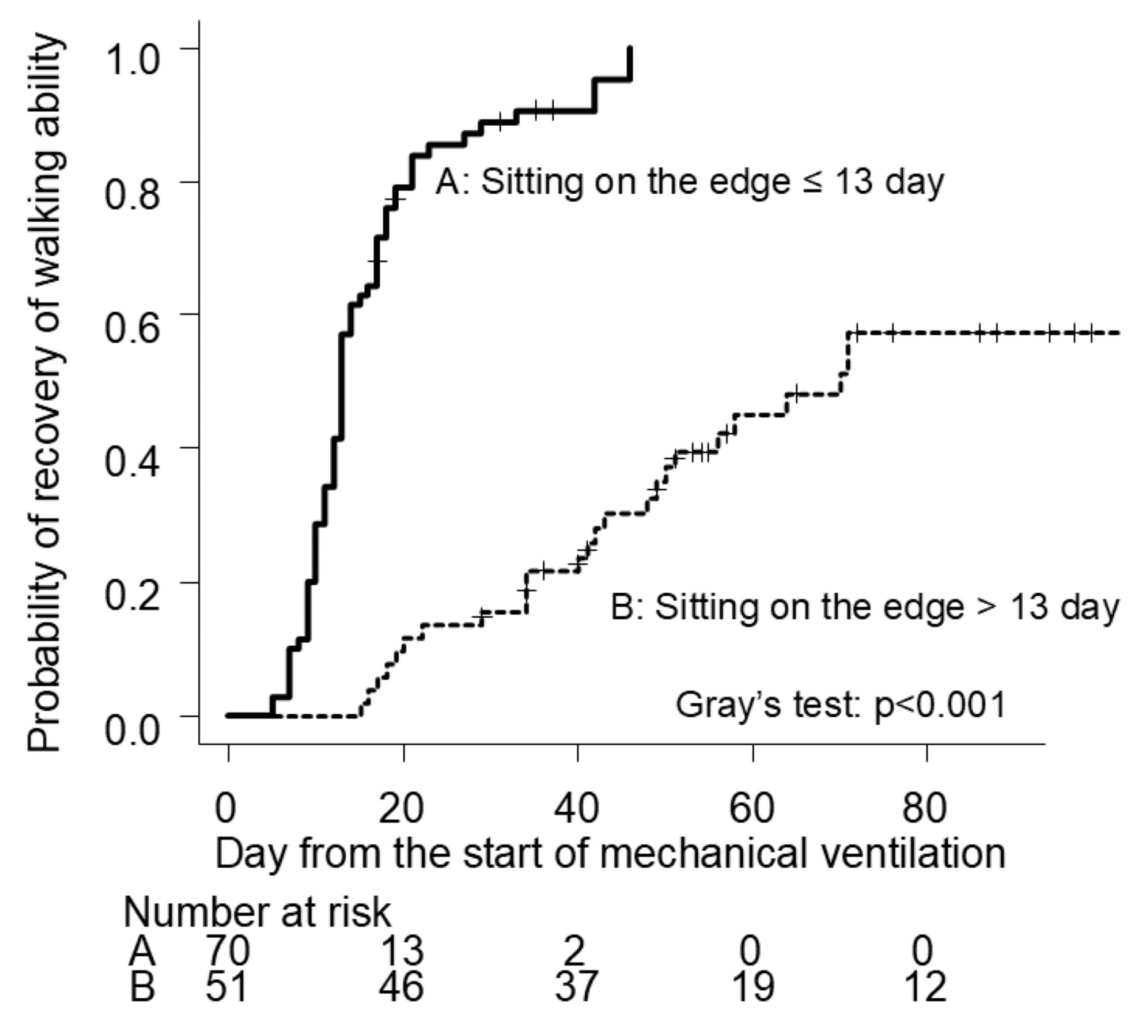
Recovery rate of walking ability between the two groups during 90 days after mechanical ventilation management

Cox proportional hazards regression analysis identified significant associations between age, duration of mechanical ventilation, and time to initiation of sitting on the edge of the bed (Table 2). Based on the ROC curve analysis, the optimal cutoff for the time to sitting initiation was determined to be 13 days, with a sensitivity of 80.0%, specificity of 70.3%, and an area under the curve (AUC) of 0.77 (95% CI: 0.68-0.86) (Figure 2). Patients who started sitting on the edge of the bed after 13 days had an adjusted hazard ratio (HR) of 0.11 (95% CI: 0.05-0.23). Gray’s test revealed a significant delay in walking recovery in the > 13-day group compared to the ≤ 13-day group (p<0.001, Figure 3).

**Table 2:** Factors affecting recovery of walking ability (FAC ≥ 3) at hospital discharge Likelihood ratio test p < 0.001, Proportional hazard assumption p= 0.21 FAC: Functional Ambulation Categories, HR: hazard ratio, CI: Confidence Interval, APACHE II: Acute Physiology and Chronic Health Evaluation, CCI: Charlson Comorbidity Index; PaO_2_: partial pressure of oxygen; FiO_2_: fraction of inspired oxygen

Variables	Univariate		Multivariate	
	HR (95%CI)	p-value	Adjusted HR (95%CI)	p-value
Age (1 yr)	0.99 (0.98-1)	0.18	0.98 (0.97-1.00)	0.016
APACHE II score (1 point)	0.95 (0.92-0.98)	0.002	1.03 (0.99-1.07)	0.11
CCI (1 point)	0.81 (0.68-0.97)	0.021	0.92 (0.79-1.08)	0.31
Duration of mechanical ventilation (1 day)	0.18 (0.11-0.30)	<0.001	0.97 (0.95-1.00)	0.049
PaO_2_/F_I_O_2_ (20 ratio)	1.05 (1.02-1.08)	<0.001	1.02 (0.98-1.05)	0.33
Hyperglycemia >200mg/dl	0.53 (0.34-0.82)	0.005	0.87 (0.54-1.39)	0.55
Sitting on the edge > 13 day	0.09 (0.05-0.17)	<0.001	0.11 (0.05-0.23)	<0.001

## Discussion

We found that delayed timing in achieving a sitting position (≥ 13 days) in critically ill ICU patients was associated with delayed recovery of walking ability (defined as FAC ≥ 3), even after adjusting for age, illness severity, and duration of mechanical ventilation. This finding may represent one clinical indicator for the timing of rehabilitation interventions aimed at improving walking recovery in critically ill patients.

ICU patients are at high risk of developing ICU-acquired weakness (ICU-AW) [25,26], which considerably delays functional recovery, including walking ability. According to Mehrholz et al., patients specifically diagnosed with ICU-AW require a median of 28.5 days to regain walking ability and approximately 81.5 days to fully recover [22]. While their study does not specifically address the timing of sitting, it underscores the detrimental effects of prolonged immobility. In our study, although sitting delay was independently associated with delayed walking recovery after adjusting for confounders, it is important to note that the duration of mechanical ventilation, which is a known contributor to ICU-AW and immobility [27,28], was also significantly longer in the non-walking group. We suggest that sitting delay may not act in isolation but rather reflect underlying illness severity and physiological instability, such as sedation requirements or respiratory dysfunction. In this context, sitting delay may serve as a clinical marker of severity rather than a direct cause of poor walking recovery.

Previous studies have emphasized that early rehabilitation significantly improves functional outcomes in ICU patients. Watanabe et al. reported that the total duration of rehabilitation activity in the ICU was an important predictor of walking independence [29]. Similarly, Hamazaki et al. demonstrated that initiating early-phase rehabilitation was associated with a higher likelihood of both walking independence and discharge to home [30]. While these studies highlight the value of early mobilization, they do not provide a specific temporal threshold for initiating key rehabilitation milestones. Our findings add to this literature by identifying a clinically relevant cutoff of 13 days for achieving a sitting position at the edge of the bed. Watanabe et al. reported that patients achieved sitting at the edge of the bed within a median of six days [6], which is notably earlier than the median of 11 days observed in our study. This discrepancy may reflect institutional differences in rehabilitation protocols or patient management practices. In our setting, factors such as mandatory DVT screening before initiating mobilization, deep sedation for respiratory management (median deep sedation period: six days in our cohort), or the absence of standardized rehabilitation and sedation protocols could have contributed to the prolonged time to sitting. However, ICU-AW, which is a potential confounding factor in functional outcomes, was not directly assessed in our study. Therefore, caution is warranted when interpreting the observed associations.

Several factors may contribute to the delayed recovery of sitting and walking in critically ill patients. It is important to note that delayed initiation of sitting is not itself the primary problem, but rather a reflection of underlying severe illness and physiological instability. Severe illnesses, such as sepsis and acute respiratory distress syndrome, increase disease severity, leading to prolonged cardiovascular and respiratory instability that limits early mobilization [9,31]. Prolonged sedation delays awakening and rehabilitation participation [32], further contributing to delays in sitting. Cardiovascular and respiratory impairments further hinder mobility [33]. Additionally, reduced physical activity and neuromuscular impairment in the ICU also play a role [34]. Our findings suggest that the timing of achieving sitting can be used as a practical benchmark to estimate the progress of rehabilitation and to guide clinical decision-making, rather than a strict target to be immediately achieved. Such a benchmark may help inform rehabilitation planning, but should be applied in safe early mobilization in each patient’s general condition.

This study had some limitations. First, we did not assess ICU-AW and delirium, which may have influenced outcomes. Second, because the study only included survivors at discharge, excluding those who died, the recovery rate of walking ability may have been overestimated. This selection bias may have excluded patients with more severe illness, underscoring the need to include non-survivors in future studies to better reflect overall recovery patterns. Third, the initiation of sitting on the edge of the bed was delayed in this study compared to previous reports, with a median of 11 days versus six days reported in earlier studies [6]. This delay may be attributable to the absence of a standardized protocol [35] and the requirement for physician judgment and lower extremity DVT screening prior to initiating sitting. Fourth, this was a single-center retrospective study, which may limit the generalizability of our findings. Causal relationships cannot be established, and observed associations should be interpreted with caution due to the potential for unmeasured confounding, including the lack of a comprehensive assessment of frailty.

## Conclusions

In this study of critically ill patients requiring prolonged mechanical ventilation, we found that delayed acquisition of a sitting position, especially when it occurred after day 13, was significantly associated with poor walking recovery at discharge. This highlights the importance of early functional milestones, such as sitting, as indicators of physical recovery. Recognizing delays in these milestones may help clinicians, including physical and occupational therapists, identify patients at risk for poor physical recovery and adjust care plans accordingly. These findings may serve as baseline data highlighting how the absence of standardized early mobilization protocols and the implementation of DVT screening can delay the initiation of sitting at the edge of the bed.
